# Do Bilinguals Acquire Similar Words to Monolinguals? An Examination of Word Acquisition and the Similarity Effect in Japanese—English Bilinguals’ Vocabularies

**DOI:** 10.3390/ejihpe11010014

**Published:** 2021-02-20

**Authors:** Aya Kutsuki

**Affiliations:** Department of Psychology, Kobe Shoin Women’s University, Kobe 657-0015, Japan; ayakutsuki@shoin.ac.jp; Tel.: +81-78-882-6122

**Keywords:** bilingual children, cross-linguistic influence, cognates, English, Japanese, katakana, profile effect, form-similarity effect, vocabulary acquisition

## Abstract

Previous research has paid much attention to the overall acquisition of vocabularies among bilingual children in comparison to their monolingual counterparts. Much less attention has been paid to the type of words acquired and the possible transfer or cross-linguistic effects of the other language on vocabulary development. Thus, this study aims to explore similarities and dissimilarities in the vocabularies of simultaneous bilinguals and Japanese monolinguals and considers the possible cross-linguistic similarity effect on word acquisition. Six simultaneous Japanese–English bilingual children (mean age = 34.75 months (2.56)) were language–age-matched with six Japanese monolinguals; their productive vocabularies were compared regarding size and categories. Additionally, characteristic acquired words were compared using correspondence analyses. Results showed that, although delayed due to the reduced inputs, young bilinguals have a similar set of vocabularies in terms of word category as monolinguals. However, bilingual children’s vocabularies reflect their unevenly distributed experience with the language. Fewer interactive experiences with language speakers may result in a lower acquisition of interactive words. Furthermore, there is a cross-linguistic effect on acquisition, likely caused by form similarity between Japanese *katakana* words and English words. Even between languages with great dissimilarities, resources and cues are sought and used to facilitate bilingual vocabulary acquisition.

## 1. Introduction

It is widely known that bilingual children can have smaller vocabularies in individual languages than their monolingual peers, but that their overall vocabulary (considering both languages together) is larger [1,2].

Notwithstanding the generally expected extra cognitive load for learning two languages, bilingual children seem to acquire two languages quite smoothly and without much hindrance. Similarly to monolingual toddlers, bilingual toddlers also start producing words in their first year of their life [3], and their vocabulary size falls within the normal range of monolingual scores [4,5]. There are reasonable concerns for educational achievements of immigrant bilingual children; therefore, the focus of many studies has been on size of their vocabularies in comparison to monolingual children. However, much less attention has been paid to differences in what comprises bilingual vocabularies and their possible differences from monolingual vocabularies.

Earlier studies came to the conclusion that the rate of vocabulary acquisition for one of the languages by bilingual children is not much different from that of monolingual children [2]. It is also well documented that when two languages are combined, the overall vocabulary of bilingual children is larger than the vocabulary of monolingual children [1,6]. However, more novel studies have shown a slower rate of vocabulary acquisition for bilinguals. In other words, when bilingual children are compared to monolingual children of the same age, they tend to have a smaller vocabulary in both languages [7,8,9,10,11].

In fact, some studies suggest that the delays remain throughout their lives because the experience of bilinguals with each language is never the same. Such effects of differential experiences with each language on the language abilities of bilinguals are known as “profile effects” [12]. That is, words tend to be more circumstance-specific for bilinguals because their vocabulary knowledge is distributed across two languages. However, studies that explore such differences in the structure of young bilingual children’s vocabularies are still scarce.

To see the effect of such distribution in the structure of vocabulary in young children, it is useful to study young bilingual children raised in double language homes wherein one of the languages is only used at home but not much outside. The child’s exposure to the language is limited, and the child experiences much fewer opportunities or circumstances to use or hear that language than a monolingual child who lives in a society where that language is mainly spoken.

The existence of the other language may also affect the word acquisition of bilinguals when compared to monolinguals. When two languages are acquired simultaneously, they are not treated as completely separate; some aspects are shared, meaning language transfers and cross-linguistic structures may be observed in bilingual children’s linguistic output [13,14,15,16,17].

The genetic and typological similarities of languages (e.g., syntactic, conceptual, lexical, and/or phonological similarities) can contribute to general cross-linguistic transfer [18]. Interaction of the vocabularies of bilingual individuals’ languages can cause a similarity effect, meaning cognates may be processed faster than non-cognates [19,20]. Hemsley et al. [18] compared the acquisition of cognates and other types of nouns in terms of conceptual and form/phonological similarities and found a cognate advantage in production when compared to other noun types, suggesting that the similarity effect plays a role in young second language (L2) learners’ vocabulary acquisition. According to the bilingual lexicon model [21,22], although the words of two languages are separate at the lexical level, they can share conceptual information. More specifically, when a sequential bilingual acquires a word in L2 for which an equivalent in his/her first language (L1) already exists, the L1 word is connected to the relevant concept in the lexicon, and the L2 word is usually directly connected to the L1 word at the representation level; the L2 word connects to the same concept mainly through the L1 word. Similarities in the representations of the words in the two languages seem to facilitate this process; the form similarity of words is likely to impact the acquisition process [23,24]. The facilitation effect of word similarity or cognates has been extensively studied in the vocabulary of adult bilinguals and second language (L2) learners. [25,26,27,28]. Such effect is also observed in school-aged bilinguals and L2 learners [29,30,31]. Studies examining the cognate facilitation effect have been typically performed on speakers or learners who are school-aged or older and are acquiring languages with close genetic proximities. However, not much is known about such effects in the vocabulary acquisition by younger children learning a pair of genetically unrelated languages.

### Form Similarity between English and Japanese

The current study explored the vocabulary of bilingual toddlers raised with both the English and Japanese languages.

Although it is still debatable, the Japanese language does not belong to any language families. There is no genetic proximity or morphological similarities between Japanese and English. However, contemporary Japanese includes words that originate from foreign languages, such as English, and are written in a syllabary called *katakana*. *Katakana* words with foreign origins phonetically resemble the sounds of the original words to varying degrees, such as ジュース/ *juuce* which means “juice” in English. Due to the phonetic resemblance, *katakana* words are treated like cognates in psycholinguistic studies, and cross-linguistic effects [31,32,33,34] and cognate/facilitation effects [35,36,37] have been observed in adult Japanese learners of the English language.

The effect of form similarities on bilingual vocabulary acquisition in very young simultaneous bilinguals has not yet been investigated. Furthermore, studies of vocabulary acquisition in simultaneous bilinguals have emphasized that such bilinguals’ overall vocabulary size is not very different from that of monolinguals, or even larger when the two languages are combined; little attention has been attributed to possible differences in the words acquired.

The research questions are formulated in (1) and (2).

(1) Does bilingual children’s limited experience with each language affect the kind of words they acquire compared to those of monolingual children?
(2) Are some words acquired differently by Japanese–English bilingual children than by Japanese monolingual children due to the cross-linguistic effect, such as the form-similarity effect?

Therefore, the current study investigates similarities and differences between the types of words acquired in Japanese by Japanese–English bilingual children and by Japanese monolingual children, and examined the possible form-similarity effects of English in Japanese vocabulary acquisition.

The rate and methods of dual language acquisition are affected by the combination and genetic proximity of the two languages [12]. Despite long years of homogeneity, Japan is now experiencing an increasing number of migrants, with children of migrant families being exposed to two languages. However, the acquisition of two language combinations, including Japanese, is still an understudied area. Studying the vocabulary acquisition of young Japanese–English bilinguals will contribute to this developing domain of research, as well as provide useful information for both parents and childcare practitioners in order to better understand and support bilingual language development.

## 2. Methods

### 2.1. Participants

Two language groups comprising bilingual and monolingual children, respectively, were recruited. All the parents of the children were college-educated, and all homes were two-parent homes at the time of investigation.

Prior to participating in the study, the rights of the participants were explained to the parents of the children as approved by the researcher’s affiliation. At the time of investigation, all monolingual children lived in Japan with their Japanese-speaking parents and the parents used only Japanese with their children. All bilingual children lived in the United States with their parents; for all bilinguals, their mother’s mother tongue was Japanese and their father’s was English. When recruiting, Japanese-speaking mothers married to English-speaking fathers from the United States were recruited by means of personal contacts. All the bilingual families in the current study lived in different regions of the United States. Unlike other immigrant communities, such as Latino families residing in the United States or French-speaking communities in Canada, there is no such community outside of Japan wherein Japanese is mainly spoken. Therefore, the generalized image of a bilingual child moving back and forth between two language communities is not applicable to the present study; thus, the community influence (i.e., societal influence) was not the main focus of the current study. The bilingual children in the current study did not receive much Japanese input from outside the home, and only mainly did so from their mothers. This kind of environment provides an opportunity to study the effects of reduced opportunities to use a language on one’s vocabulary acquisition, as well as the interaction of knowledge of the two languages in acquisition.

More details regarding the bilingual children’s language environment and language use on a typical weekday are shown in Table 1. Some of the bilingual children, bi 1, bi 2, bi 5, and bi 6, received some Japanese input outside their homes, because their parents decided to send them to Japanese or Japanese and English preschools and arranged playdates with other Japanese-speaking children. However, monolingual children in Japan would hear and use Japanese more frequently compared to these bilingual children with limited opportunities with the language. Overall, 12 children, aged 24–36 months, participated; a bilingual group: six children, aged approximately 30–34 months (mean = 34.75 months (2.56)); and a monolingual group: six Japanese monolinguals, selected from a dataset collected for a previous study (mean age = 26.78 months (2.40)).

#### Vocabulary Questionnaire

The Japanese version of the MacArthur-Bates Communicative Development Inventory (JCDI): Words and Sentences [38] was answered by the parents of both the bilingual and monolingual children.

### 2.2. Procedure

Vocabulary data collection: using the JCDI, the bilingual children’s productive vocabularies were measured every 2–4 months over a period of 8–14 months. The parents received questionnaires by mail and were asked to mail them back after answering. Age and vocabulary data for both groups are summarized in Table 2 and Table 3, respectively. To form a bilingual dataset, data sourced when the bilingual children were aged 30–36 months were used. For the bilingual samples, the mean age was 34.75 months (SD = 2.56), the mean number of words produced was 402.65 months (SD = 25.27), and the mean vocabulary age was 32.50 months (SD =1.87). To conduct a comparison, data for six Japanese monolingual children were selected from a previous study which was conducted only with monolingual children. These data were chosen such that both language groups had approximately the same vocabulary size. Average vocabulary ages and numbers of words did not differ significantly between the groups (n.s. for both). For the monolingual dataset, the mean age was 26.78 months (2.40), the mean number of words produced was 396.67 months (SD = 34.67), and the mean vocabulary age was 32.67 months (SD = 1.63). There was a significant difference regarding average age: monolinguals (M = 26.78 months (2.40)) were younger than the bilinguals (M = 34.75 months (2.56)).

Two analyses were conducted in this study. Analysis 1 compared bilinguals and monolinguals regarding the types of words acquired in terms of the number of words in each category and the size of each word category. Analysis 2 compared bilinguals and monolinguals regarding the characteristic words, with a focus on individual words. All data used for the analysis came from the JCDI questionnaires.

For Analysis 1, the respective numbers of productive words were compared between groups for each of the 24 JCDI word categories.

For Analysis 2, to determine the characteristics of the two groups’ productive words, correspondence analyses were conducted on each category using 10 ordinary nouns (Categories B–K). The reason for choosing only ordinary nouns was that other word classes, such as adjectives and verbs, could be morphosyntactically and conceptually difficult to acquire and could be more strongly affected by age-specific factors than by actual language experiences. To examine the form-similarity effect of the *katakana* words in comparison to the non-*katakana* words, the former were differentiated and written in italics in the tables and figures for the analysis. When English words are adopted into the Japanese language, their sounds (and occasionally meanings) become modified; this causes some *katakana* words to differ greatly from their original meanings in English; however, we focused on the fact that they (or at least some parts of them) originate from English words, regardless of similarities in meaning. Additionally, words that do not directly come from English but that resemble their corresponding words were also included as *katakana* words (e.g., *koppu* means “cup” but was originally adopted from Dutch).

To identify the characteristics of the types of words used by each group, for each category, data were summarized in a contingency table, with the rows indicating individual words and the columns representing individual children from the two groups. Then, through correspondence analysis for which Multivariate Analytic System Seagull-Stat was used, the dimensions were interpreted based on the associations between the row- and column-based categories of the contingency table (individual words and language groups). Therefore, where and how words are plotted and the constituents of the language-group clusters could demonstrate the similarities of their relationships, which would show the characteristics of words acquired by each child.

## 3. Results and Discussions

### 3.1. Analysis 1

Analysis 1 investigated the first research question; whether limited experience with each language affects bilingual children’s vocabulary and whether the kind of words they acquire differ from those acquired by monolingual children.

Nonparametric comparisons (Mann–Whitney U test) revealed no significant differences in the numbers of productive words for all categories except C (vehicles), V (games and routines), and W (baby words II) (U = 5.00, *p* < 0.05; U = 3.50, *p* < 0.05; U = 4.50, *p* < 0.05, respectively; Table 4).

The groups had almost identical numbers of words from each category in their respective vocabularies, suggesting similar acquisition; thus, less exposure to Japanese does not create a great difference in the kinds of word acquired in general, but bilingual children require more time to acquire the same kind of vocabulary set as monolingual children. However, the bilinguals in this study were approximately eight months older, implying that bilinguals needed eight months to catch up with the monolinguals in most of the word categories.

There was no great difference between the kinds of words acquired by the groups except for a few types of words, because the mothers in the current study stayed at home and looked after their children and spoke almost entirely in Japanese with the child, except for a few activities and occasions outside the home and when their father talked. Therefore, as far as the bilingual children in this study were concerned, there was almost no uneven distribution of experience with the Japanese language. They were acquiring almost the same set of vocabulary, only more slowly due to the reduced inputs. If the bilingual children had more opportunities to use English, such as going to school where English is spoken or spending time with English speakers, their daily experience would have been more greatly divided across the two languages and their vocabularies would have been unevenly distributed; in such a scenario more prominent profile effects may have been observed.

However, a few group differences were observed across three categories: C (vehicles), M (games and routines), and V (baby words II). Here, the focus is on categories M and V, because the bilinguals acquired fewer words than the monolinguals in these categories. The words in these two categories are not simple words (i.e., nouns) but are instead words used in interactions with people. Examples of such words in Category M are *dozo* (“here you are”), *oshimai* (“finish”), and *chodai* (“gimme”); meanwhile, many terms in Category V, such as *choki* (‘”cutting sound”), *chun* (“chirping sound”), and *guru* (“spinning sound”) are the baby forms of onomatopoeic words, which are frequently used by native Japanese speakers when talking with small children. Furthermore, although the bilinguals were older, they still had a lower number of interactive words than the monolinguals, implying that they needed even more time to acquire them. One may wonder why only interactive words needed more time for acquisition compared to the rest of the words. A cross-cultural study [39] indicated that when talking with their infants, Japanese mothers use more baby forms and place a greater emphasis on interaction with people than American mothers. Although there is no study on the child-directed speech of Japanese adults, many are familiar with the style and use it to speak with young children. The fact that the bilinguals in the current study did not live in a Japanese speaking community and they had very limited interaction with Japanese-speaking people may have caused lower and possibly slower acquisition of “interactive words”, such as those in Categories M and V. Another possible reason for the delayed or lower acquisition of interactive words is time limitation. It is widely known that the older the child, the less parents and adults use the baby forms of Japanese with them.The average age of the bilinguals was 36 months; people would still use the baby forms but maybe to a lesser extent than with younger children. In this sense, the baby forms may have been used for a relatively limited time window, rendering them more susceptible to reduced inputs while other kinds of words were heard and picked up anytime later in life. 

Contrasting with Categories M and V, for Category C, the bilinguals had significantly more words than the monolinguals. This could be because there were more *katakana* words (Japanese words that originate from foreign languages) in this category, which caused a similarity effect. This aspect is examined in Analysis 2. 

These results provide tentative answers to the first research question: Does bilingual children’s limited experience with each language affect the kind of words they acquire compared to those of monolingual children? The answer is only partly yes. Based on the results, one could suggest that even with only one Japanese-speaking parent and with fewer opportunities to use Japanese outside the family than if they had been in Japan, the bilinguals achieved a similar vocabulary set as the monolinguals, although they took more time due to the reduced inputs. However, the acquisition of some kinds of words is more strongly affected by the experience of using the language with people, and young bilingual children who have limited inputs were more susceptible to this effect. These findings reflect the previously discussed “profile effects” [12].

### 3.2. Analysis 2

All the figures for correspondence analyses and the lists of vocabularies for each word category are included in the supplementary data.

The analyses demonstrated two distinct patterns, named here Patten 1 and Pattern 2. Figures S1–S10 show the correspondence analyses results, where bi 1–bi 6 represent each bilingual child, and mono 1–mono 6 represent each monolingual child; the other plots indicate individual words in the children’s productive vocabularies. Note that words from the categories that were not produced are included in the tables but are not plotted in the figures. The four quadrants in this analysis are Quadrants I (in the upper right), II (upper-left), III (bottom-left), and IV (bottom-right).

For each category, a table containing words in Japanese (with katakana words in italics) and their translations is provided (Tables S1 to S10) in the supplementary data.

Pattern 1 demonstrates two distinct characteristics of the groups, specifically due to the bilingual children clustering within two neighboring quadrants. This pattern is present for Categories B (animals), C (vehicles), E (food and drink), F (clothing), H (furniture and items in the house), and I (small household items).

For the word categories that indicated Pattern 1, the two groups showed differing acquisition patterns. For example, for Category B, in Figure S1, the five bilingual children were clustered together in Quadrant III, while three monolingual children were in Quadrant II. Examining the words surrounding these clusters shows that, for the bilinguals in Quadrant III, words such as *hato* (“pigeon”), *nezumi* (“mouse”), *kitsune* (“fox”), *ushi* (“cow”), and *tanuki* (“raccoon”) are present, while the monolingual cluster in Quadrant II is surrounded by *neko* (“cat”), *inu* (“dog”), *kame* (“turtle”), and *sakana* (“fish”). The words surrounding the bilingual children were the names of animals children would not see in everyday life and are more sophisticated, while the words surrounding the monolingual cluster were names of animals that are more common and are simpler to acquire. Therefore, the division between the groups may be due to the age difference, because the older bilingual children would have had more opportunities to see rare animals. For Category C, in Figure S2, the bilingual children seemed to cluster in Quadrants II and III, while the monolinguals tended to be located in Quadrants I and IV. In Quadrant III, words such as *torakku*, *baiku*, and *herikoputa* were plotted closer to the three bilingual children, while in Quadrant II, words such as *danpuka*, *shobosha*, and *patoka* were plotted close to three bilingual children and one monolingual child.

The four monolinguals in Quadrants I and IV were relatively far apart, indicating that they did not share much similarity. Moreover, the words were not very close to them, meaning that these monolingual children acquired no characteristic words in this category. The words close to the bilinguals were mostly *katakana* words (in italics), which represent English words that have been modified and adopted into the Japanese language. This means that words that somewhat resemble English words are more characteristic of the bilinguals’ vocabulary than the monolinguals’ vocabulary. A similar pattern can also be seen in Categories E, F, H, and I, where English-derived *katakana* words clustered around the bilinguals (see Tables S3–S6 and Figures S3–S6).

In Patten 2, there was no obvious differentiating pattern between the two groups; children from both groups were spread over at least three quadrants. This pattern existed for Categories D (toys), G (body parts), J (outside things), and K (places to go).

Examination of the correspondence-analysis data for Category D (Figure S7) showed that both bilinguals and monolinguals were spread over three quadrants, indicating no clear tendency in either group. This may mean the words surrounding each child concerned individual characteristics regarding word acquisition rather than group tendency. This pattern was also observed for Categories G, J, and K (see Tables S8–S10 and Figures S8–S10).

It is interesting that these four categories have a very low percentage of *katakana* words (Table 5). This suggests that *katakana* words are acquired somewhat differently by Japanese monolinguals when compared to Japanese–English bilinguals. However, this also raises the question of whether *katakana* words are more difficult to acquire than non-*katakana* words.

### 3.3. Are Katakana Words More Difficult to Acquire Than Other Nouns?

As explained in the Methods section, in order to compare the kinds of words acquired by monolingual and bilinguals, in the current study, we language–age-matched the two groups, which created a significant difference (approximately eight months) between their respective chronological ages (bilinguals mean = 34.75 months (2.56), and monolinguals mean = 26.78 months (2.40)). Thus, this age difference and the increased difficulty of *katakana* words may have created the abovementioned observed differences.

Although there has been no previous examination of the difficulty children experience acquiring *katakana* words such as those used in this study, comparing the difficulty of acquiring *katakana* words with that for other nouns is useful here. Using comprehensive acquisitional data for (monolingual) Japanese children (*n* = 2861), obtained using JCDI data that were previously collected for standardization purposes [40], we measured the percentages of children who acquired certain individual words at ages 26 and 34 months (these ages were closest in this previous study to the average ages of the two groups in the current study). If *katakana* words are more difficult to acquire than non-*katakana* words, the percentage of children with individual *katakana* words in their vocabulary should be smaller than those with non-*katakana* words. To conduct this comparison, we used the average attainment rates (average percentage of children acquiring individual words in each category) between the *katakana* and non-*katakana* words. For each category, the average percentage of attainment rate of individual *katakana* words and non-*katakana* words at 24 and 36 months were calculated and compared using *t*-tests (Table 6). Category G was excluded from this analysis because it does not contain any *katakana* words.

As Table 6 shows, only Categories J and K demonstrated significant differences between *katakana* and non-*katakana* words, as well as in attainment rates (averages of percentage of children who acquired each word in the dataset used for standardization [40]) at the two ages of 26 months and 34 months. In fact, for these categories, at both ages, more children know non-*katakana* than *katakana* words. The fact that these two categories concern “going out” may have caused the age difference, because older children tend to have more opportunities to explore outside.

The overall trend suggests that, for children of approximately this age, *katakana* nouns are generally not more difficult to acquire than other nouns, which implies that the observed differences between the groups were not due to age differences but the effect of the existence of the other language (i.e., English) in the bilinguals’ word acquisition.

Additionally, we compared the two groups regarding the number of *katakana* and non-*katakana* words (Table 7), and only found a significant difference regarding the *katakana* words, not the non-*katakana* words. This further suggests the *katakana* words are acquired somewhat differently by bilinguals compared to monolinguals.

Analysis 2 considered nouns acquired by individual children from both groups. Two patterns for characteristic words acquired were identified when the two groups’ vocabularies were concurrently analyzed, demonstrating group and individual differences, respectively. The former suggests a possible influence of simultaneous acquisition of English. *Katakana* words, which originated from and phonetically resembled corresponding English words, were characteristic of bilinguals’ vocabulary but not for monolinguals.

Several studies have reported that form similarity between bilinguals’ two languages influences translation equivalents (TE) acquisition. More specifically, phonological similarities contribute to the early acquisition of TEs. Schelletter [24] studied German-English bilingual children’s language development and reported that such children show more advanced acquisition of form-similar words than form-dissimilar words between the two languages. Meanwhile, Bosch et al. [23] proposed the facilitation effect of form similarity; if the words of two languages are very similar, the words’ frequency of use can double, and they can be more easily learned than non-similar words. They also suggested that the similarity effects can extend to similar-sounding words. Based on these and the current results, *katakana* words can act as cognates in English–Japanese bilingual acquisition. However, one must be careful with the degree of similarity of *katakana* words. Although *katakana* words mostly have some resemblance with their original words in foreign languages, their sounds are modified to fit the Japanese phonology and are not as close as those of the cognates in European languages. In fact, they are so distant that English instructors and learners in Japan generally believe that *katakana* words have a negative influence on English pronunciation. *Katakana* words are unintelligible to non-Japanese speakers and often become obstacles when trying to speak in English [41]. Conversely, they did not have such negative effects on vocabulary acquisition of the bilinguals in the current study. This is probably because the bilingual children had some sort of metalinguistic awareness of phonetic similarities and dissimilarities of the two languages and could interpret some sounds that might be very different from their original sounds as variations or similar sounds to those of the original sounds. The positive effects of such phonological awareness or knowledge on English word learning are partially supported by evidence from adult L2 English learners [35,36,37]. These studies demonstrate that Japanese adults learning English, who are aware of both the phonetical similarities and dissimilarities of the *katakana* words with their original words, can treat them as cognates to some extent. Furthermore, L2 studies [42,43] argue that teaching Japanese students such similarities and dissimilarities has a positive effect on their English vocabulary learning. It seems that in L2 learning, the links between the words are enhanced by such knowledge and by forming pairs or cognates across the languages. It is widely known that bilingual children have better metalinguistic awareness or ability than their monolingual counterparts [44,45]; therefore, it is reasonable to assume that English–Japanese bilingual children use their primitive awareness when acquiring *katakana* words. Along with the primitive awareness of such phonetic properties, bilingual-specific sound sensitivity may also play a role. Infants are extremely sensitive to the phonetic properties of words and 15-month-olds only accept word labels (word sounds) in their native accent; however, with increasing exposure to the language, at around 19 months, they accept some variations in pronunciation [46,47]. In a similar vein, Ramon-Casas et al. [48] compared the abilities of Catalan–Spanish bilingual children and Catalan monolingual children to distinguish minor phonological variations in cognates. It was found that the bilingual children had to be older, at least three and half years old, to detect changes in sounds that two-year-old monolinguals were able to. This suggests that bilingual children build language specific phonetic recognition later than monolinguals. Although little is known about how form-similar words are recognized and treated across a pair of distant languages with few phonological similarities, findings from previous studies and the current results suggest that the sounds of *katakana* words and those of their original English words may have been treated as variations of each other in the vocabulary development of the English–Japanese bilingual children. Children in the current study were almost three years old; therefore, the effects of cognitive or phonetic (in)sensitivity or less language specific phonetic representation might have started to diminish, and the *katakana* words might have been acquired differently because of such cognitive processing. On the other hand, metalinguistic awareness is well underway for three-year-olds. The remnant effects of both phonetic insensitivity and metalinguistic ability are likely to be reflected in the word acquisition patterns seen in this study.

For very young English–Japanese bilingual children, the links between *katakana* words with varying degrees of phonetical similarities with their original English words are already being created; moreover, they are acquired and organized differently from other non-*katakana* words in their vocabulary development process.

Furthermore, based on the facilitation effects of cognates observed in word acquisition of young bilinguals [29,30,31], one can reasonably expect English–Japanese bilingual children to acquire *katakana* words faster than non-*katakana* words. However, additional research will be needed to prove this point. 

The current results demonstrate the existence of a cross-linguistic effect of form similarity in Japanese–English bilingual vocabulary acquisition and may provide an affirmative answer to the second research question: “Are some words acquired differently by Japanese–English bilingual children compared to Japanese monolingual children due to cross-linguistic effects, such as the form-similarity effect?” Furthermore, the current results also suggest that such an effect can manifest in developing vocabularies with less linguistic proximity, which has not been demonstrated previously.

### 3.4. Limitations

There are some limitations to this study. Firstly, the sample size was limited. It was a small study conducted by a single researcher; therefore, only six bilingual children who were around the same age and raised in a similar language environment were recruited. To match the bilingual group, only six participants were recruited for the monolingual group as well. For this small sample size, the author was forced to mostly use nonparametric methods; however, this provided an excellent opportunity to examine individual words in close detail.

Secondly, there was an age difference between the groups. Ideally, this age difference would have been smaller to avoid the age effect. However, in reality, it was only possible to either create groups of the same age but with different language levels or of differing ages and identical language levels; this is because bilinguals tend to trail monolinguals in proficiency in a single language. For the purpose of the current study, which was to closely consider the kinds of words bilingual children acquire in relation to their monolingual counterparts, language-matching was appropriate. Lastly, the present study did not control or compare the degree of similarities between *katakana* words and their original words, because it is difficult to define the former. The effects of form similarity may differ depending on such similarities and should be considered in future studies.

## 4. Conclusions

The primary aims of this study were to explore in-depth similarities and dissimilarities in the Japanese vocabularies of bilinguals with limited exposure to the Japanese language and those of monolinguals, and to examine the possibility of a cross-linguistic effect in terms of form similarity. Our results provide preliminary answers in this regard. Overall, young bilinguals and monolinguals have similar vocabularies regarding word categories, except in certain categories which are more susceptible to the amount of input; meanwhile, there is a form-similarity effect regarding the acquisition of certain types of words when acquiring a pair of very distant languages: Japanese and English.

Bilingual acquisition (i.e., Japanese and another language) by very young children is still an understudied area in bilingual research. With the increasing migrant population in Japan, empirical data on bilingual acquisition are valuable, which can provide necessary information for parents, educators, and researchers alike to better support and understand bilingual acquisition in Japan.

## Figures and Tables

**Table 1:** The bilingual (bi) children’s language environments.

Language Use at Home and Outside	bi 1	bi 2	bi 3	bi 4	bi 5	bi 6
**Parents mother tongues**						
Mother’s mother tongue	Japanese	Japanese	Japanese	Japanese	Japanese	Japanese
Father’s mother tongue	English	English	English	English	English	English
**Places stayed, duration (hours) and languages used**						
Languages used at home	English Japanese	English Japanese	English Japanese	English Japanese	English Japanese	English Japanese
Hours spent at home (awake)	9	6	9	4	8	8
Places outside home	Preschool	Kindergarten	Preschool	Preschool	Outside, playdates	Preschool
Language used outside home	English Japanese	Japanese	English	English	English Japanese	Japanese
Hours spent outside home	1–2	6	8	8	4	1
**Percentage (%) of each language used by Mother to the child**						
English (%)	20	0	0	5	2	1
Japanese (%)	80	100	100	90	98	99
**Percentage (%) of each language used by Father to the child**						
English (%)	95	100	95	40	100	100
Japanese (%)	5	0	5	40	0	0
**Average hours spent with the child (hours)**						
Mother alone	8	9	9	4	6	8
Father alone	1	1	0	1	1	2
Both parents	1	1	4	1	1	2

**Table 2:** Age and vocabulary data for the bilingual children.

ID	bi 1	bi 2	bi 3	bi 4	bi 5	bi 6	Mean	SD
Gender	female	female	female	male	male	male		
Chronological age (months)	36.10	39.17	32.00	34.13	33.00	34.07	34.75	2.56
Number of words	389	405	427	422	359	414	402.67	25.27
Vocabulary age (months)	30	31	32	34	33	35	32.50	1.87

**Table 3:** Age and vocabulary data for the monolingual (mono) children.

ID	Mono 1	Mono 2	Mono 3	Mono 4	Mono 5	Mono 6	Mean	SD
Gender	female	female	female	male	male	male		
Chronological age (months)	24.93	26.63	30.96	26.93	24.00	27.20	26.78	2.40
Number of words	385	436	421	336	398	404	396.67	34.67
Vocabulary age (months)	30	34	32	32	34	34	32.67	1.63

**Table 4:** Comparison between the bilingual and monolingual children’s respective vocabularies regarding the numbers of words in each word category of the Japanese version of the MacArthur-Bates Communicative Development Inventory (JCDI).

Categories	Total Number of Words	Monolinguals	(SD)	Bilinguals	(SD)	Comparison between Bilinguals and Monolinguals
A. Baby word I	12	8.83	(73.61)	5.67	(47.22)	n.s.
B. Animals	43	24.83	(57.75)	34.67	(80.62)	n.s.
C. Vehicles	**14**	**7.00**	**(50.00** **)**	**11.33**	**(80.95** **)**	**mono < bi, U = 5.00, *p* < 0.05**
D. Toys	18	8.50	(47.22)	10.17	(56.48)	n.s.
E. Food and drink	68	45.33	(66.67)	45.50	(66.91)	n.s.
F. Clothing	28	10.67	(38.10)	10.67	(38.10)	n.s.
G. Body parts	27	21.50	(79.63)	19.33	(71.60)	n.s.
H. Furniture and rooms	33	12.17	(36.87)	14.17	(42.93)	n.s.
I. Small household items	50	25.67	(51.33)	23.50	(47.00)	n.s.
J. Outdoor items	31	12.83	(41.40)	15.33	(49.46)	n.s.
K. Places to go	22	8.17	(37.12)	10.50	(47.73)	n.s.
L. People	29	16.00	(55.17)	14.83	(51.15)	n.s.
M. Games and routines	**25**	**23.83**	**(95.33** **)**	**21.33**	**(85.33** **)**	**mono > bi, U = 3.50, *p* < 0.05**
N. Action words	103	74.17	(72.01)	65.00	(63.11)	n.s.
O. Time	12	3.83	(31.94)	4.00	(33.33)	n.s.
P. Descriptive words	63	37.33	(59.26)	42.00	(66.67)	n.s.
Q. Pronouns	22	6.67	(30.30)	8.67	(39.39)	n.s.
R. Question words	10	3.67	(36.67)	4.67	(46.67)	n.s.
S. Locations	26	4.50	(17.31)	4.83	(18.59)	n.s.
T. Quantifiers and articles	17	9.17	(53.92)	11.50	(67.65)	n.s.
U. Connecting words	6	0.67	(11.11)	5.17	(86.11)	n.s.
V. Baby word II	**29**	**18.33**	**(63.22)**	**8.83**	**(30.46)**	**Mono > bi, U = 4.50. *p* < 0.05**
W. Conversational words	14	7.33	(52.38)	5.50	(39.29)	n.s.
X. Other	9	5.67	(62.96)	5.50	(61.11)	n.s.
Total	711	396.67	(50.89)	402.67	(54.91)	n.s.
Note. Significant group difference are indicate in bold letters and numbers.						

**Table 5:** Percentages of *katakana* words in categories B–K.

JCDI Word Category	B Animals	C Vehicles	D Toys	E Food and Drink	F Clothing	G Body Parts	H Furniture and Rooms	I Small House-Hold Items	J Outside Things	K Places to Go
Total number of words	43	14	18	68	28	27	33	50	31	29
English-derived *Katakana*	5	6	7	28	17	0	13	17	2	7
% of *Katakana* words	12%	43%	39%	41%	61%	0%	39%	34%	6%	24%

**Table 6:** Comparisons between bilinguals and monolinguals regarding the attainment rates of words.

JCDI Word Category	Age in Months	Type of Words	Number of Words in Each Category	Attainment Rates	SD	*t*	*df*	*t*-Test	*p*-Value
B	26	Non-*Katakana*	38	53.41	16.92	−0.37	41.00	n.s.	0.71
		*Katakana*	5	56.30	10.87				
	34	Non-*Katakana*	38	79.79	14.88	−0.34	41.00	n.s.	0.73
		*Katakana*	5	82.16	11.71				
C	26	Non-*Katakana*	8	59.84	15.82	0.86	12.00	n.s.	0.41
		*Katakana*	6	51.70	19.62				
	34	Non-*Katakana*	8	83.50	11.14	1.93	12.00	n.s.	0.08
		*Katakana*	6	69.38	16.31				
D	26	Non-*Katakana*	11	44.35	16.90	1.56	16.00	n.s.	0.14
		*Katakana*	7	30.23	21.44				
	34	Non-*Katakana*	11	79.55	14.18	2.20	16.00	n.s.	0.04
		*Katakana*	7	63.26	17.07				
E	26	Non-*Katakana*	41	55.44	20.83	−1.71	66.00	n.s.	0.09
		*Katakana*	27	46.99	18.50				
	34	Non-*Katakana*	41	82.60	15.98	−0.78	66.00	n.s.	0.44
		*Katakana*	27	79.62	14.76				
F	26	Non-*Katakana*	10	45.52	29.83	−1.49	26.00	n.s.	0.15
		*Katakana*	18	30.32	23.50				
	34	Non-*Katakana*	10	64.12	33.68	−0.75	26.00	n.s.	0.46
		*Katakana*	18	55.48	26.27				
H	26	Non-*Katakana*	20	27.67	22.50	−0.61	31.00	n.s.	0.55
		*Katakana*	13	32.36	20.66				
	34	Non-*Katakana*	20	60.84	22.86	−0.26	31.00	n.s.	0.80
		*Katakana*	13	62.96	22.84				
I	26	Non-*Katakana*	32	40.88	21.00	−0.44	48.00	n.s.	0.56
		*Katakana*	18	37.98	24.53				
	34	Non-*Katakana*	32	68.29	23.70	−0.71	48.00	n.s.	0.48
		*Katakana*	18	69.92	23.37				
J	26	Non-*Katakana*	29	36.48	23.41	0.86	29.00	*p* < 0.01	0.40
		*Katakana*	2	21.90	20.79				
	34	Non-*Katakana*	29	64.64	25.39	0.19	29.00	*p* < 0.01	0.85
		*Katakana*	2	61.05	43.91				
K	26	Non-*Katakana*	15	37.86	21.91	−5.01	17.25	*p* < 0.01	0.00
		*Katakana*	7	7.66	5.45				
	34	Non-*Katakana*	15	71.15	25.84	−0.44	20.00	*p* < 0.01	0.00
		*Katakana*	7	25.63	10.73				

**Table 7:** Comparison between bilinguals and monolinguals regarding numbers of *katakana* and non-*katakana* words.

Word Types	Language Groups	*n*	Average Number of Words Acquired	SD	*t*	*df*	*t*-Test	*p*-Value
*Katakana*	Bilingual	6	61.67	10.57	−3.01	10	<bi	−0.26
	Monolingual	6	47.17				<bi	
Non-*Katakana*	Bilingual	6	133.50	22.66	−0.22	10	n.s.	−0.22
	Monolingual	6	130.17				n.s.	

## Supplementary Materials

The following are available online at https://www.mdpi.com/2254-9625/11/1/14/s1, Table S1–S10 and Figure S1–S10 for the results of the correspondence analyses.
