# Supplementary material for: Do Bilinguals Acquire Similar Words to Monolinguals? An Examination of Word Acquisition and the Similarity Effect in Japanese—English Bilinguals’ Vocabularies
Source: Eur J Investig Health Psychol Educ. 2021 Feb 20;11(1):168–82. doi: 10.3390/ejihpe11010014 (PMC8314333; doi:10.3390/ejihpe11010014)
Supplement: Supplementary file 1 [file ejihpe-11-00014-s001.pdf]

Table S1. The word list with English translations for category B (animals)

no.	JCDI words	Romanaized	English translation
b1	あひる	ahiru	duck
b2	あり	ari	ant
b3	犬	inu	dog
b4	うさぎ	usagi	bunny
b5	牛	usi	cow
b6	うま	uma	horse
b7	えび	ebi	shrimp/prawn
b8	おおかみ	okami	wolf
b9	かえる	kaeru	frog
b10	かに	kani	crab
b11	かば	kaba	hippopotamus
b12	かめ	kame	turtle
b13	からす	karasu	craw
b14	きつね	kitsune	fox
b15	きりん	kirin	giraffe
b16	くじら	kujira	whale
b17	くま	kuma	bear
b18	コアラ	*koara*	koala
b19	ゴリラ	*gorira*	gorilla
b20	さかな	sakana	fish
b21	さる	saru	monkey
b22	しまうま	simauma	zebra
b23	ぞう	zou	elephant
b24	たぬき	tanuki	raccoon dog
b25	ちょうちょ	choucho	buttefly
b26	どうぶつ	doubutsu	animal
b27	とら	tora	tiger
b28	とり	tori	bird
b29	にわとり	niwatori	chicken
b30	ねこ	neko	cat
b31	ねずみ	nezumi	mouse
b32	はち	hachi	bee
b33	はと	hato	pigeon
b34	パンダ	*panda*	panda
b35	ひつじ	hitsuji	lamb

b36	ひよこ	hiyoko	chick
b37	ぶた	buta	pig
b38	へび	hebi	snake
b39	ペンギン	*pengin*	penguin
b40	虫	mushi	bug
b41	ライオン	*raion*	lion
b42	りす	risu	squirrel
b43	わに	wani	alligator

Table S2. The word list with English translations for category C (vehicles)

no.	JCDI words	Romanaized	English translation
c1	汽車	kisya	train
c2	車（自動車）	kuruma	car
c3	三輪車	sanrinsha	tricycle
c4	自転車	jitensha	bicycle
c5	消防車	shobosha	firetruck
c6	ダンプカー	danpuka	damp truck
c7	電車	densha	train
c8	トラック	*torakku*	truck
c9	バイク	*baiku*	motocycle
c10	バス	*basu*	bus
c11	パトカー	patoka	police car
c12	飛行機	hikoki	airplane
c13	船	fune	boat
c14	ヘリコプター	*herikoputa*	helicopter

Table S3. The word list with English translations for category E (food and drink)

no.	JCDI words	Romanaized	English translation	no.	JCDI words	Romanaized	English translation
e1	アイスクリーム	*aisukurimu*	ice cream	e35	チーズ	*chizu*	cheeze
e2	あめ（飴）	ame	candy	e36	チョコレート	*chokoreto*	chocolate
e3	いちご（苺）	ichigo	strawberry	e37	とうふ（豆腐）	tofu	tofu
e4	うどん	udon	noodles	e38	ドーナツ	*donatsu*	donut
e5	おいも	oimo	potato	e39	トマト	*tomato*	tomato
e6	お菓子	okashi	candy	e40	なし（梨）	nashi	pear
e7	お砂糖	sato	sugar	e41	なす	nasu	egg plant

e8	おすし	osushi	sushi	e42	なっとう（納豆）	nattou	firmented beans (natto)
e9	おにぎり・おむすび	onigiri	rice ball	e43	肉	niku	meat
e10	お茶	ocha	tea	e44	にんじん	ninjin	carrot
e11	柿	kaki	persimon	e45	のり（海苔）	nori	seaweed (nori)
e12	かぼちゃ	kabocha	pumpkin	e46	バター	*bata*	butter
e13	ガム	*gamu*	gum	e47	バナナ	*banana*	banana
e14	カレー	*kare*	curry	e48	パン	*pan*	bread
e15	牛乳	gyunyu	milk	e49	ハンバーガー	*hanbaga*	hamburger
e16	きゅうり	kyuri	cucumber	e50	ハンバーグ	*hanbagu*	salisbury steak
e17	クッキー	*kukki*	cokie	e51	ピーマン	piman	green pepper
e18	ケーキ	*keki*	cake	e52	ぶどう	budou	grapes
e19	コーヒー	*kohi*	coffe	e53	プリン	*purin*	caramel custard/ flan
e20	こおり（氷）	kori	ice	e54	ほうれん草	horensou	spinach
e21	コーン	*kon*	corn	e55	ポッキー	pokki	pokky (product name)
e22	ごはん（お米のこと）	gohan	rice	e56	ホットケーキ	hottokeki	pankake
e23	さかな	sakana	fish	e57	ポテト（チップス）	*poteto*	french fries
e24	サンドイッチ	*sandoicchi*	sandwich	e58	豆	mame	beans
e25	ジャム	*jyamu*	jam/jelly	e59	みかん	mikan	orange
e26	ジュース	*jyusu*	juice	e60	水	mizu	water
e27	しょうゆ	shoyu	soy sauce	e61	みそしる	misoshiru	miso soup
e28	すいか	suika	watermelon	e62	ミルク	*miruku*	milk
e29	スープ	*supu*	soup	e63	メロン	*meron*	melon
e30	スパゲティー	*supagetti*	supaghetti	e64	もも	momo	peach
e31	ゼリー	*zeri*	jello	e65	やさい	yasai	vegetable
e32	せんべい	senbei	rice cracker	e66	ヨーグルト	*yoguruto*	yogurt
e33	大根	daikon	Japanese radish	e67	ラーメン	ramen	noodles
e34	たまご	tamago	egg	e68	リンゴ	ringo	apple

Table S4. The word list with English translations for category F (clothes)

no.	JCDI words	Romanaized	English translation
f1	エプロン	*epuron*	apron
f2	オーバー	*oba*	coat

f3	おむつ	omutsu	diaper
f4	くつ	kutsu	shoe
f5	くつ下	kutsushita	sock
f6	ジーパン	jipan	jeans
f7	シャツ	*shatsu*	shirt
f8	ジャンパー	*jyanpa*	jacket
f9	スカート	*skato*	skirt
f10	ズック	*zukku*	sneakers
f11	ズボン	*zubon*	pants
f12	スリッパ	*surippa*	slippers
f13	セーター	*seta*	sweater
f14	ソックス	*sokkusu*	sock
f15	タイツ	*taitsu*	tights
f16	チャック	chakku	zipper
f17	てぶくろ	tebukuro	gloves
f18	長ぐつ	nagagutsu	boots
f19	ネクタイ	nekutai	tie
f20	ネックレス	*nekkuresu*	necklace
f21	パジャマ	*pajama*	pajamas
f22	パンツ	*pantsu*	underpants
f23	ベルト	*beruto*	belt
f24	ぼうし	bousi	hat
f25	ボタン	botan	button
f26	マフラー	*mafura*	scarf
f27	洋服	yofuku	wertern clothes
f28	リボン	*ribon*	ribbon

Table S5. The word list with English translations for category H (furniture and room)

no.	JCDI words	Romanaized	English translation
h1	いす	isu	chair
h2	エアコン	*eakon*	air-conditioner
h3	おしいれ	oshiire	closet
h4	おふろ(風呂場)	ofuro	bathroom
h5	おまる	omaru	potty
h6	カーテン	*katen*	curtain
h7	階段	kaidan	stairs

h8	げたばこ（げた箱）	getabako	shoe closet
h9	玄関	genkan	entrance
h10	こたつ	kotatsu	heater (kotatsu)
h11	シャワー	*shawa*	shower
h12	水道	suidou	tap/faucet
h13	ストーブ	*sutobu*	heater
h14	扇風機	senpuki	fan
h15	台所	daifokoro	kitchen
h16	たたみ	tatami	mats (tatami)
h17	たんす	tansu	closet
h18	駐車場	chushajyo	garage
h19	机	tsukue	desk
h20	テーブル	*teburu*	table
h21	テレビ	*terebi*	TV
h22	電気	denki	light
h23	ドア	*doa*	door
h24	トイレ	*toire*	toilet
h25	ドライヤー	*doraiya*	dryer
h26	ピアノ	*piano*	piano
h27	ひきだし	hikidasi	drawer
h28	ビデオ	*bideo*	video
h29	ベッド	*beddo*	bed
h30	部屋	heya	bedroom
h31	窓	mado	window
h32	冷蔵庫	reizoko	refregerator
h33	レンジ	*renji*	microwave oven

Table S6. The word list with English translations for category I (small household items)

no.	JCDI words	Romanaized	English translation	no.	JCDI words	Romanaized	English translation
i1	アイロン	*airon*	iron	i26	ティッシュ	*tissyu*	tissue
i2	絵	e	picture	i27	テープ	*tepu*	tape
i3	お金	okane	money	i28	電池	denchi	battery
i4	お皿	osara	dish	i29	でんわ（電話）	denwa	telephone
i5	かぎ（鍵）	kagi	keys	i30	時計	tokei	watch
i6	かご	kago	basket	i31	ナイフ	*naifu*	knife
i7	かさ（傘）	kasa	unbrella	i32	なべ	nabe	pot

i8	かばん	kaban	bag	i33	バケツ	*baketsu*	bucket
i9	かみ（紙）	kami	paper	i34	はこ（箱）	hako	box
i10	カメラ	*kamera*	camera	i35	はさみ	hasami	scisoors
i11	缶	kan	can	i36	はし（箸）	hasi	chopsticks
i12	くぎ	kugi	nail	i37	歯ブラシ	haburashi	toothbrush
i13	くし	kushi	comb	i38	ハンカチ	*hankachi*	handkerchief
i14	くすり	kusuri	medicine	i39	びん	bin	bottle
i15	コップ	*koppu*	cup/glass	i40	フォーク	*foku*	fork
i16	ごみ箱	gomi	trash	i41	ふとん	futon	
i17	財布	saifu	purse	i42	フライパン	*furaipan*	fryingpan
i18	シャンプー	*shanpu*	shampoo	i43	ほうき	hoki	broom
i19	ストロー	*sutoro*	straw	i44	包丁	hocho	knife
i20	スプーン	*supun*	spoon	i45	ポット	potto	kettle
i21	石鹸	sekken	soap	i46	マイク	*maiku*	mic
i22	ぞうきん	zokin	cloth	i47	枕	makura	pillow
i23	そうじ機	soujiki	vacuum	i48	めがね	megane	glasses
i24	タオル	*taoru*	towel	i49	めざまし	mezamashi	alarm clock
i25	茶わん	chawan	bowl	i50	毛布	mofu	blanket

Table S7. The word list with English translations for category D (toys)

no.	JCDI words	Romanaized	English translation
d1	絵本	ehon	picture book
d2	えんぴつ	enpitsu	pencil
d3	おもちゃ	omocha	toy
d4	クレヨン	*kureyon*	crayon
d5	ゲーム	*gemu*	game
d6	シャボン玉	shabondama	bubbles
d7	たいこ	taiko	drum
d8	つみき	tsumiki	block
d9	人形	ningyo	doll
d10	粘土	nendo	play dough
d11	のり（糊）	nori	glue
d12	パズル	*pazuru*	puzzel
d13	バット	*batto*	bat
d14	風船	fusen	balloon
d15	プレゼント	*purezento*	present
d16	ペン	*pen*	pen

d17	ボール	*boru*	ball
d18	本	hon	book

Table S8. The word list with English translations for category G (body parts)

no.	JCDI words	Romanaized	English translation
g1	あご	ago	chin
g2	足	ashi	leg
g3	あたま	atama	head
g4	おしり	oshiri	buttocks
g5	おチンチン	ochinchin	penis
g6	おっぱい	oppai	breast
g7	おでこ	odeko	forehead
g8	おなか	onaka	tummy
g9	おへそ	oheso	belly button
g10	顔	kao	face
g11	肩	kata	shoulder
g12	かみ（髪）	kami	hair
g13	くち	kuchi	mouth
g14	くび	kubi	neck
g15	けが	kega	mouth
g16	しっぽ	shippo	tail
g17	背中	senaka	back
g18	つめ	tsume	nail
g19	手	te	hand
g20	歯	ha	tooth
g21	鼻	hana	nose
g22	舌（べろ）	shita	tongue
g23	ほっぺ	hoppe	cheek
g24	まゆ・まゆ毛	mayu/mayuge	eyebrow
g25	耳	mimi	ear
g26	目	me	eye
g27	指	yubi	finger

Table S9.　The word list with English translations for category J (outdoor items)

no.	JCDI words	Romanaized	English translation
j1	雨	ame	rain

j2	石	ishi	stone
j3	岩	iwa	rock
j4	枝	eda	stick
j5	お月さん	otsukisan	moon
j6	お天気	otenki	good weather
j7	おひさま	ohisama	sun
j8	風	kaze	wind
j9	川	kawa	river
j10	木	ki	tree
j11	草	kusa	grass
j12	雲	kumo	cloud
j13	スコップ	sukoppu	shovel
j14	砂場	sunaba	sandbox
j15	すべり台	suberidai	slide
j16	空	sora	sky
j17	庭	niwa	backyard
j18	はしご	hashigo	ladder
j19	旗	hata	flag
j20	はっぱ	happa	leaf
j21	花	hana	flower
j22	プール	*puru*	pool
j23	ブランコ	buranko	swing
j24	ベランダ	beranda	balcony
j25	ホース	*hosu*	hose
j26	星	hoshi	star
j27	水まき	mizumaki	watering
j28	道	michi	street
j29	屋根	yane	roof
j30	雪	yuki	snow
j31	雪だるま	yukidaruma	snowman

Table S10. The word list with English translations for category K (places to go)

JCDI words	Romanaized	English translation
いえ（家）	ie	house
海	umi	beach
映画	eiga	movie
おうち	ouchi	home

お仕事	osigoto	work
おそと	osoto	outside
お店	omise	store
会社	kaishya	company/work
ガソリンスタンド	*gasorinsutando*	gas station
学校	gakkou	school
キャンプ	*kyanpu*	camping
公園	kouen	park
サーカス	*sakasu*	circus
スーパー	*supa*	supermarket
デパート	*depato*	department store
動物園	dobutsuen	zoo
ピクニック	*pikunikku*	picnic
保育所	hoikusho	preschool/nursery
森	mori	woods
山	yama	mountain
幼稚園	youchien	kindergarten / pre-school
レストラン	*resutoran*	restaurant

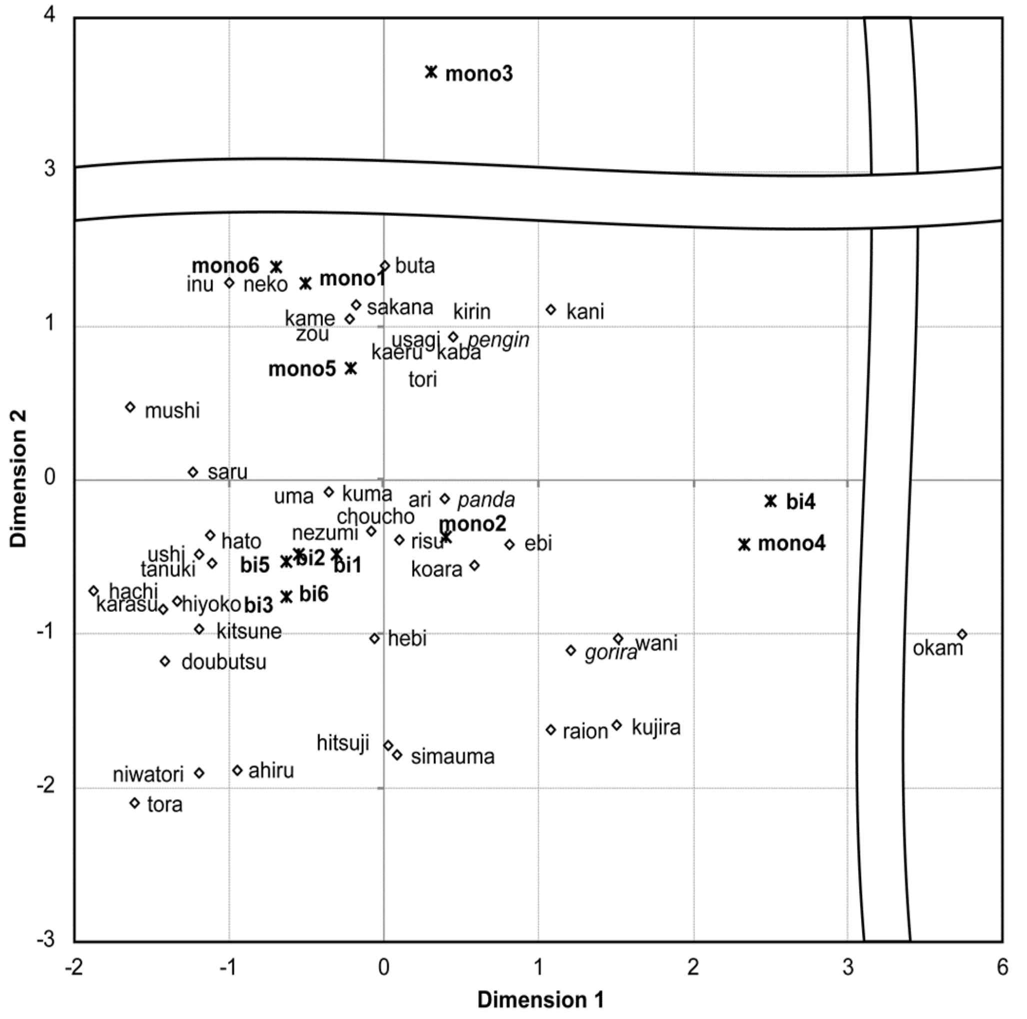

Figure S1. Characteristics of productive words in category B (animals) for bilinguals and monolinguals

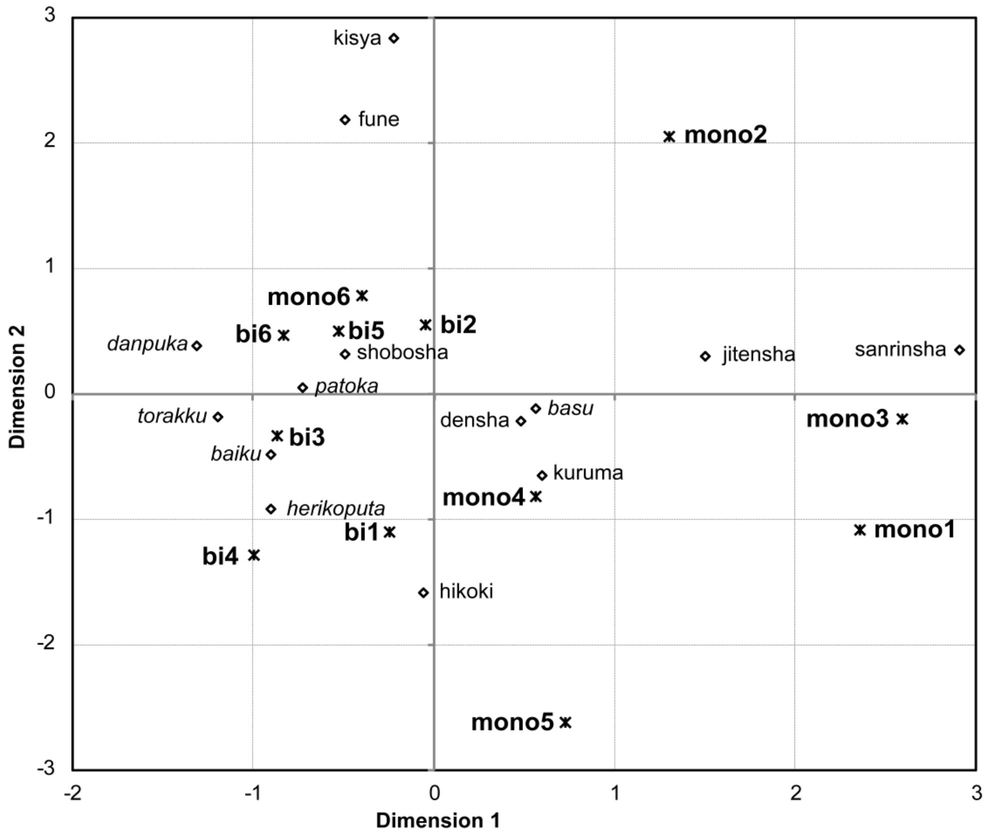

Figure S2. Characteristics of productive words in category C (vehicles) for bilinguals and monolinguals

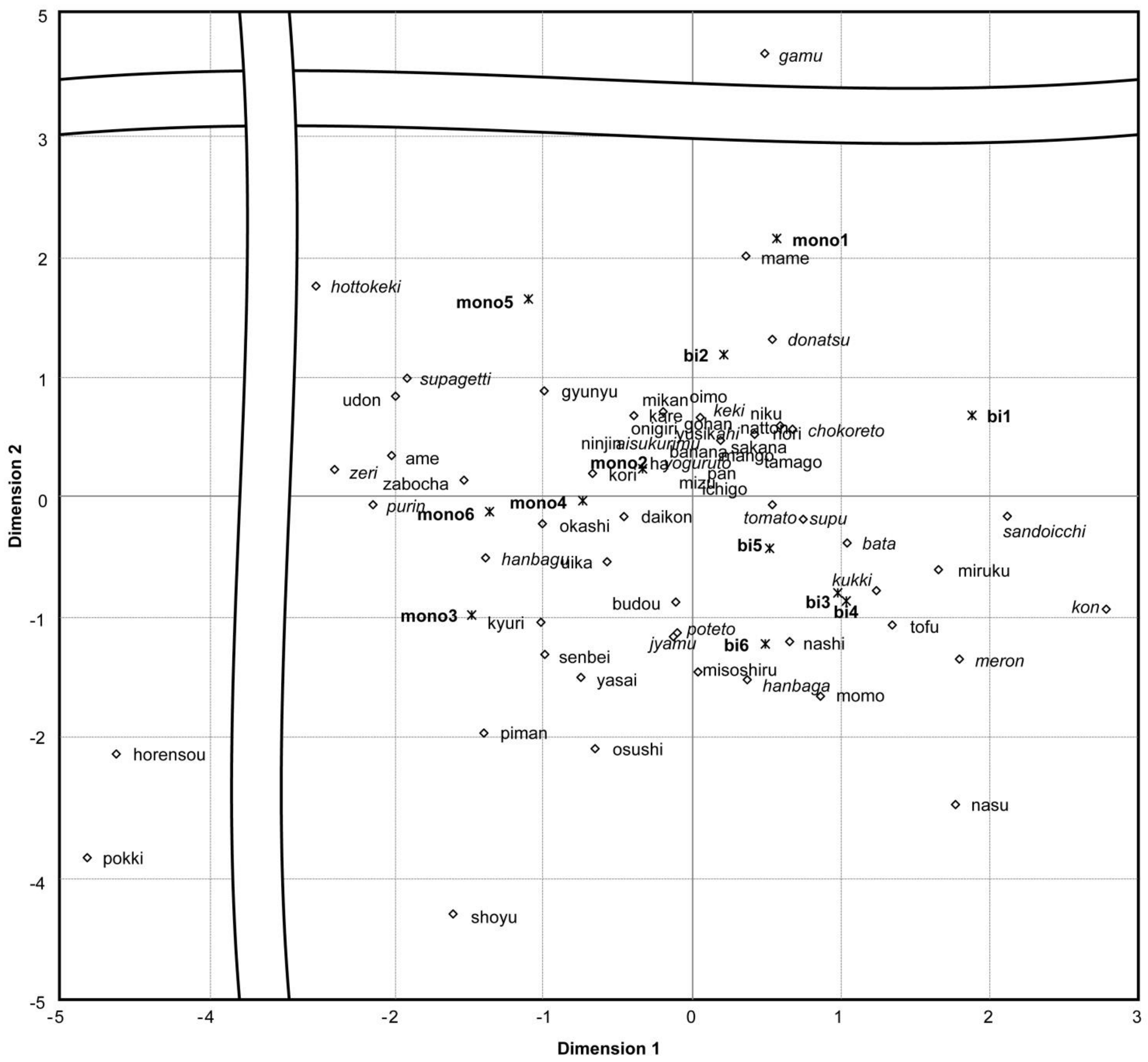

Figure S3. Characteristics of productive words in category E (food and drink) bilinguals and monolinguals

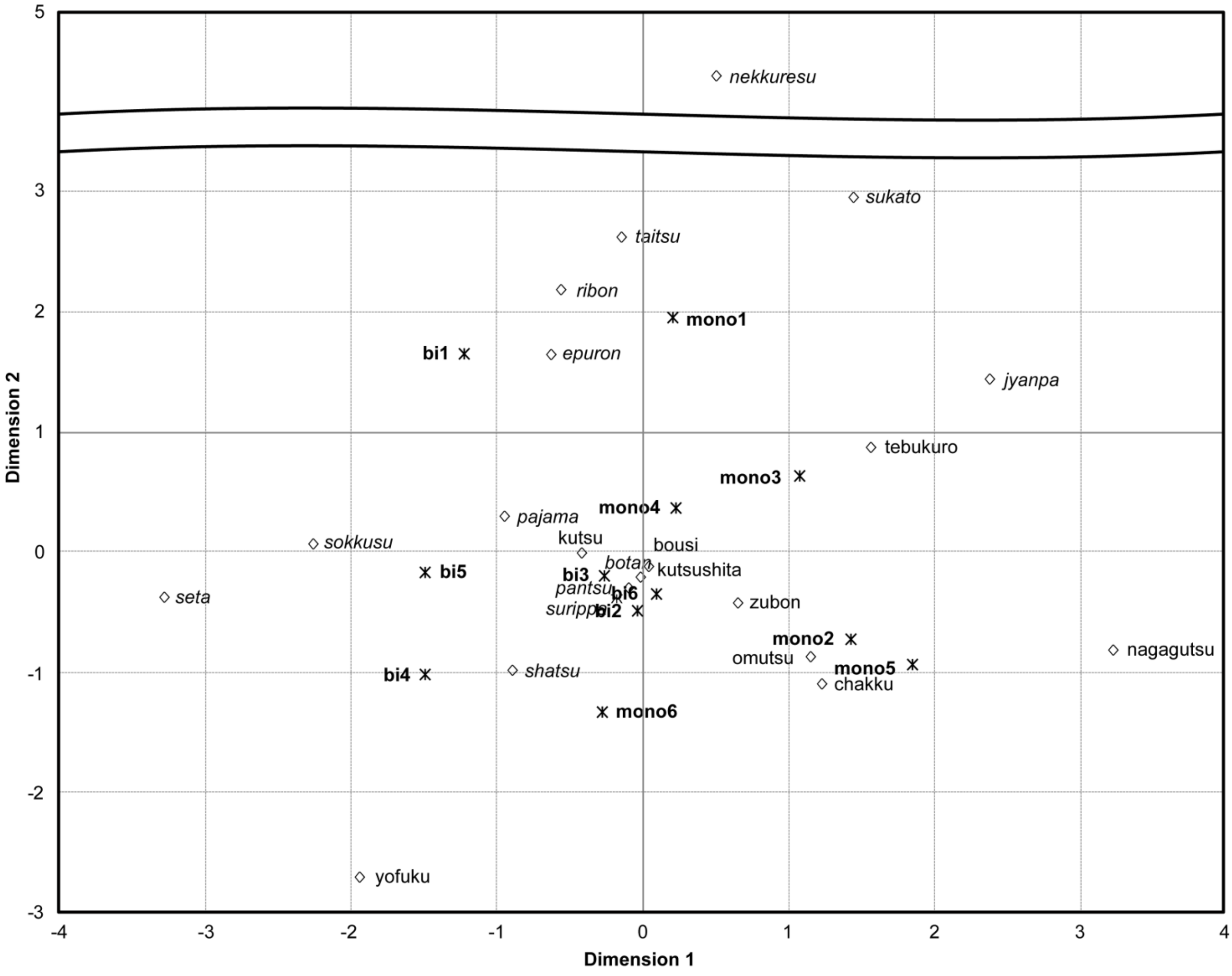

Figure S4. Characteristics of productive words in category F (clothes) for bilinguals and monolinguals

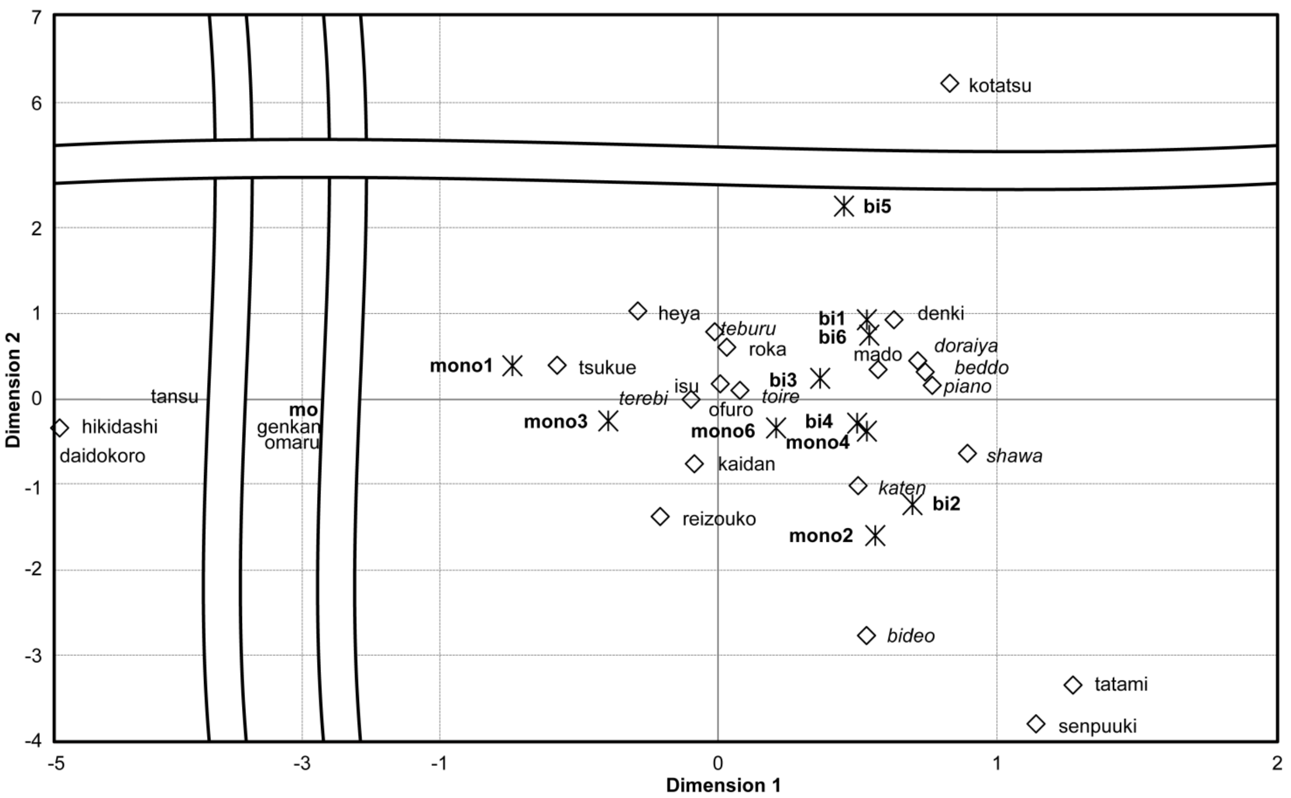

Figure S5. Characteristics of productive words in category H (furniture and room) for bilinguals and monolinguals

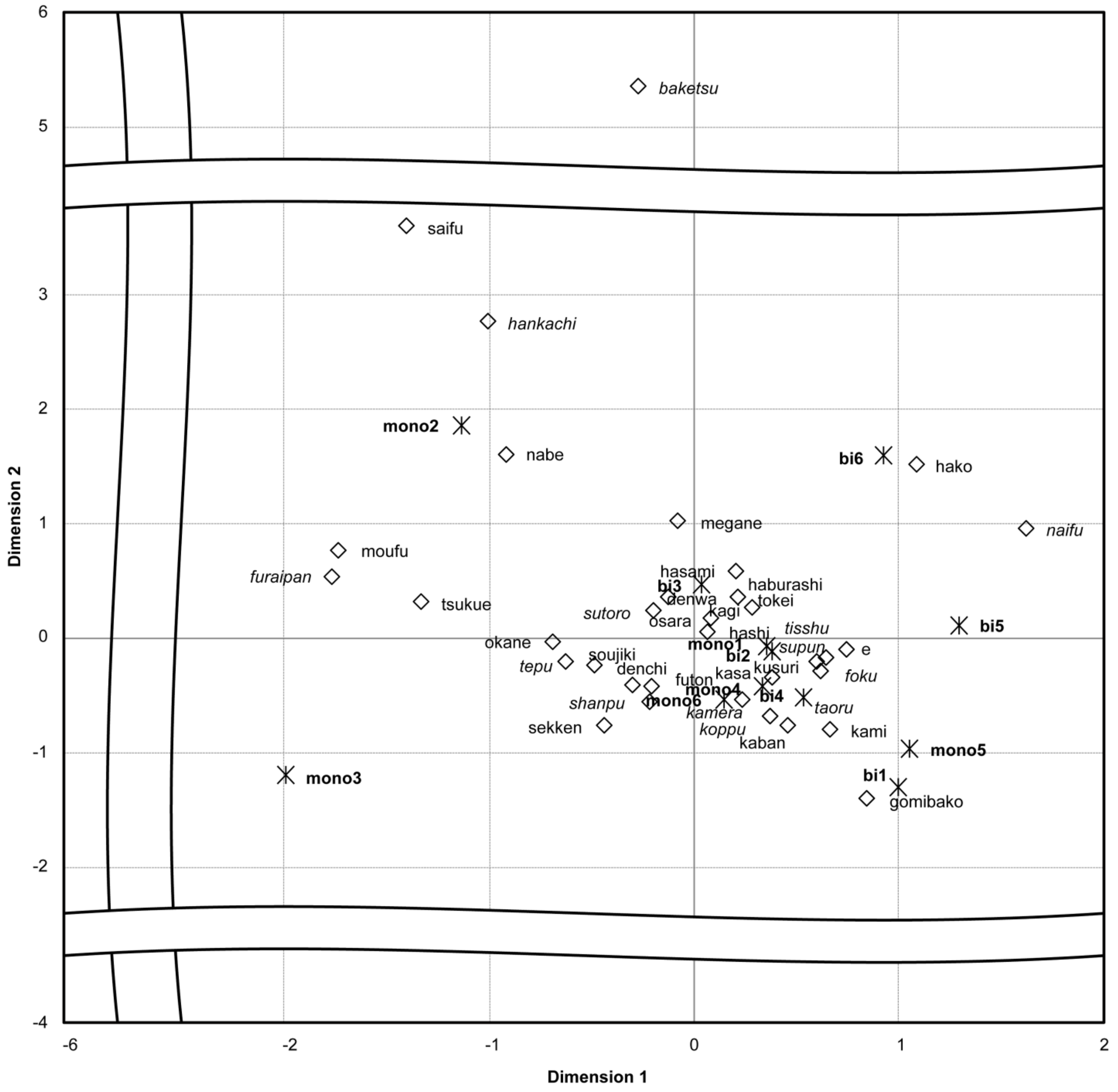

Figure S6. Characteristics of productive words in category I (small household items) for bilinguals and monolinguals

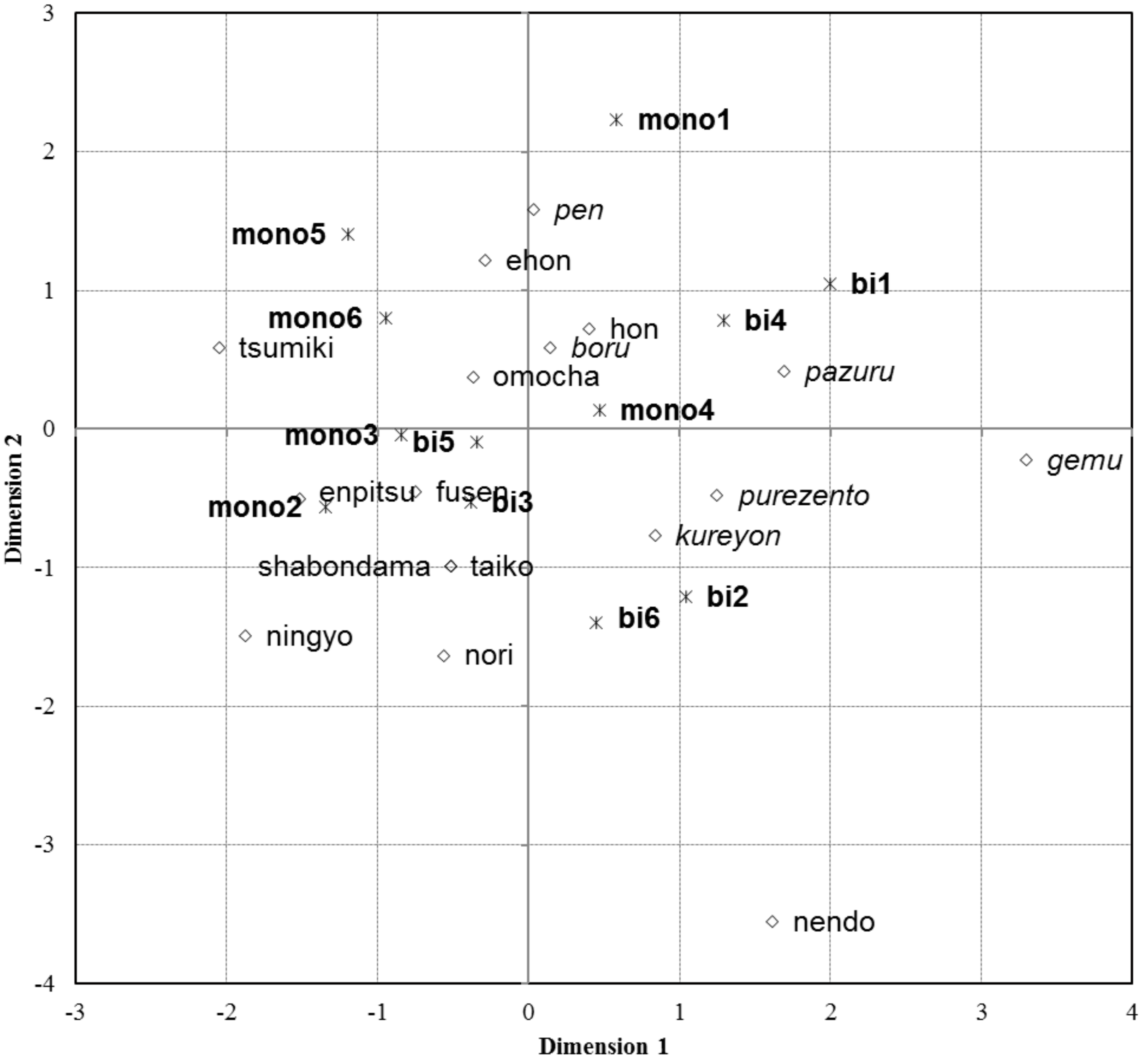

Figure S7. Characteristics of productive words in category D (toys) for bilinguals and monolinguals

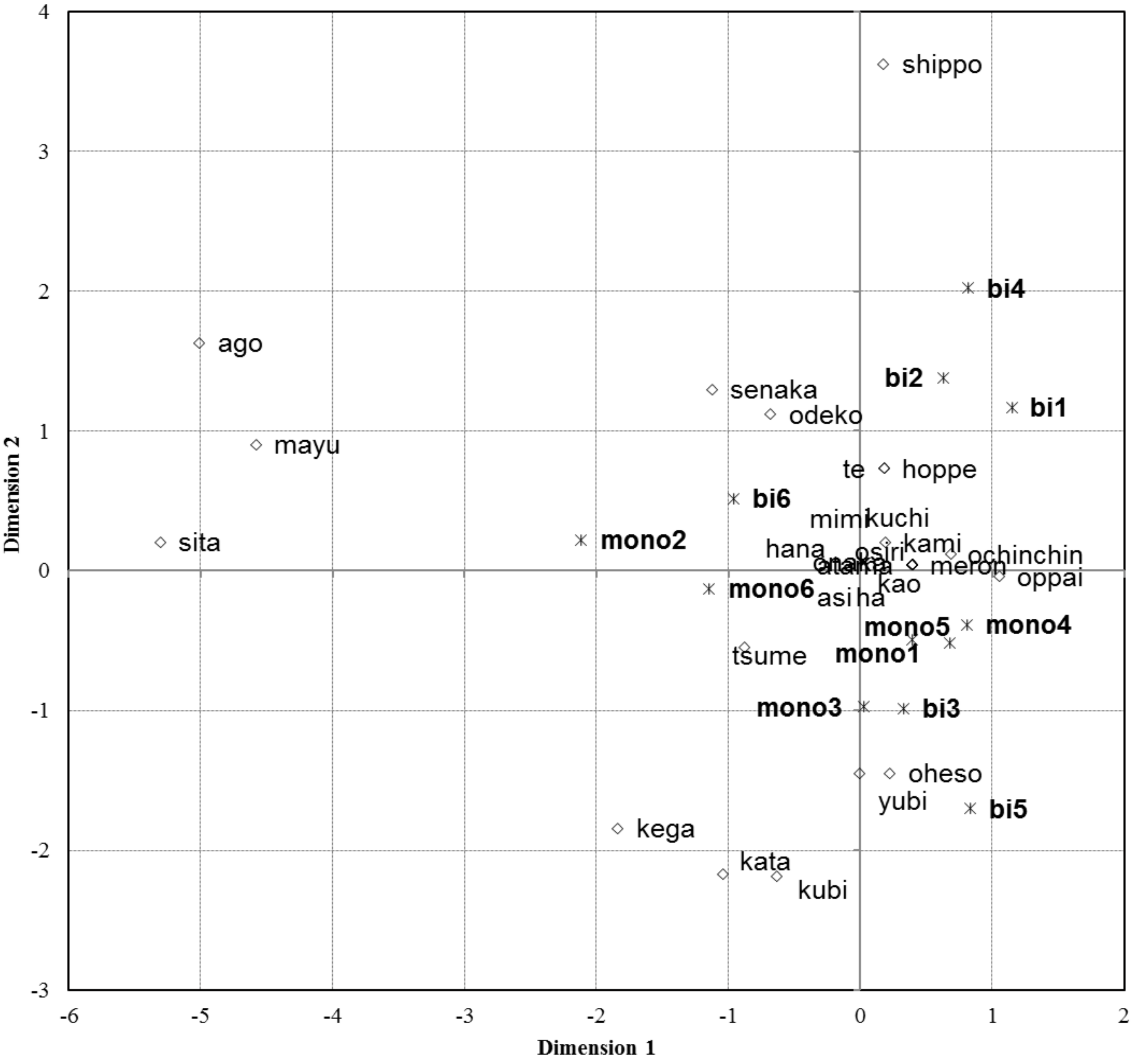

Figure S8. Characteristics of productive words in category G (body parts) for bilinguals and monolinguals

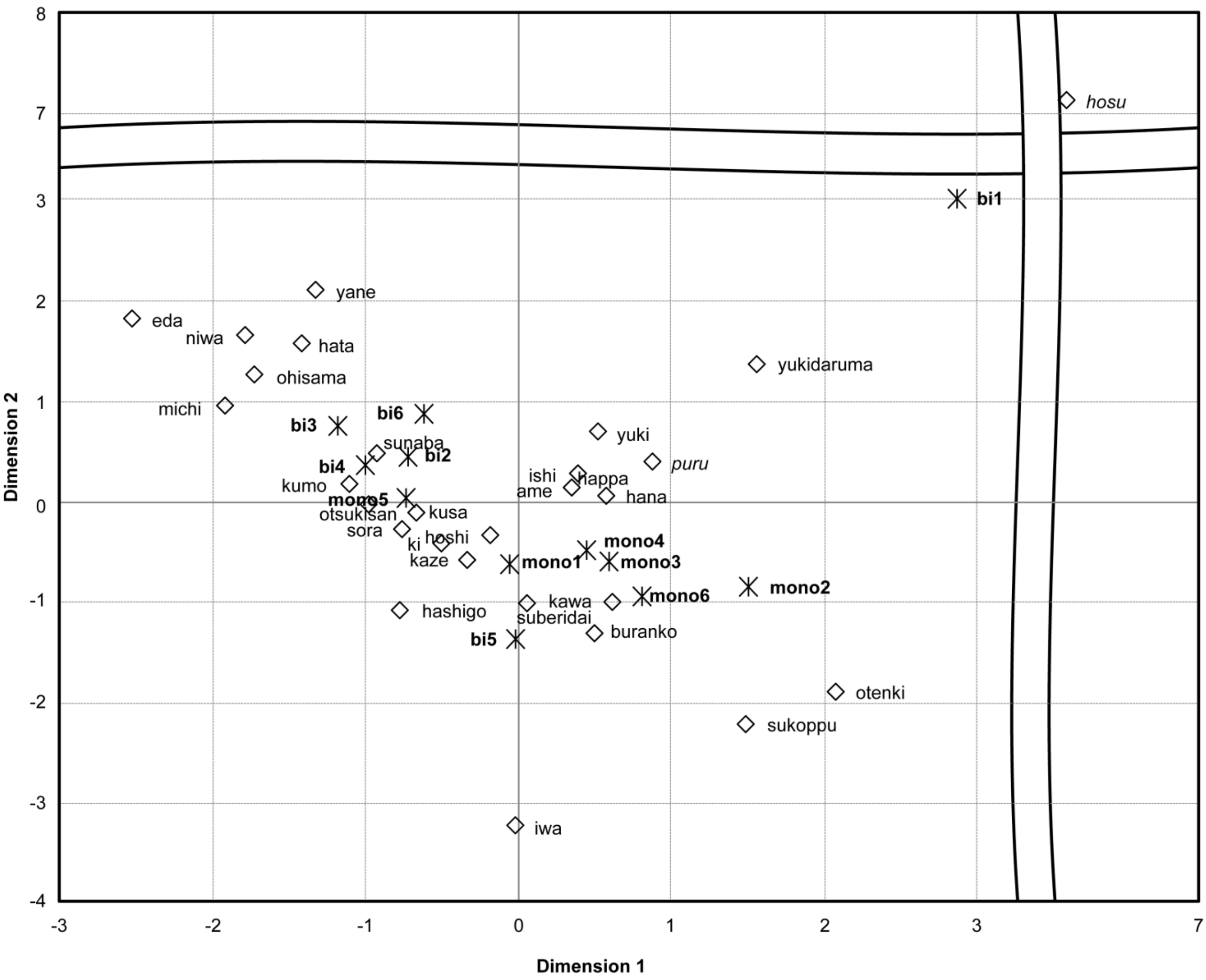

Figure S9. Characteristics of productive words in category J (outdoor items) for bilinguals and monolinguals

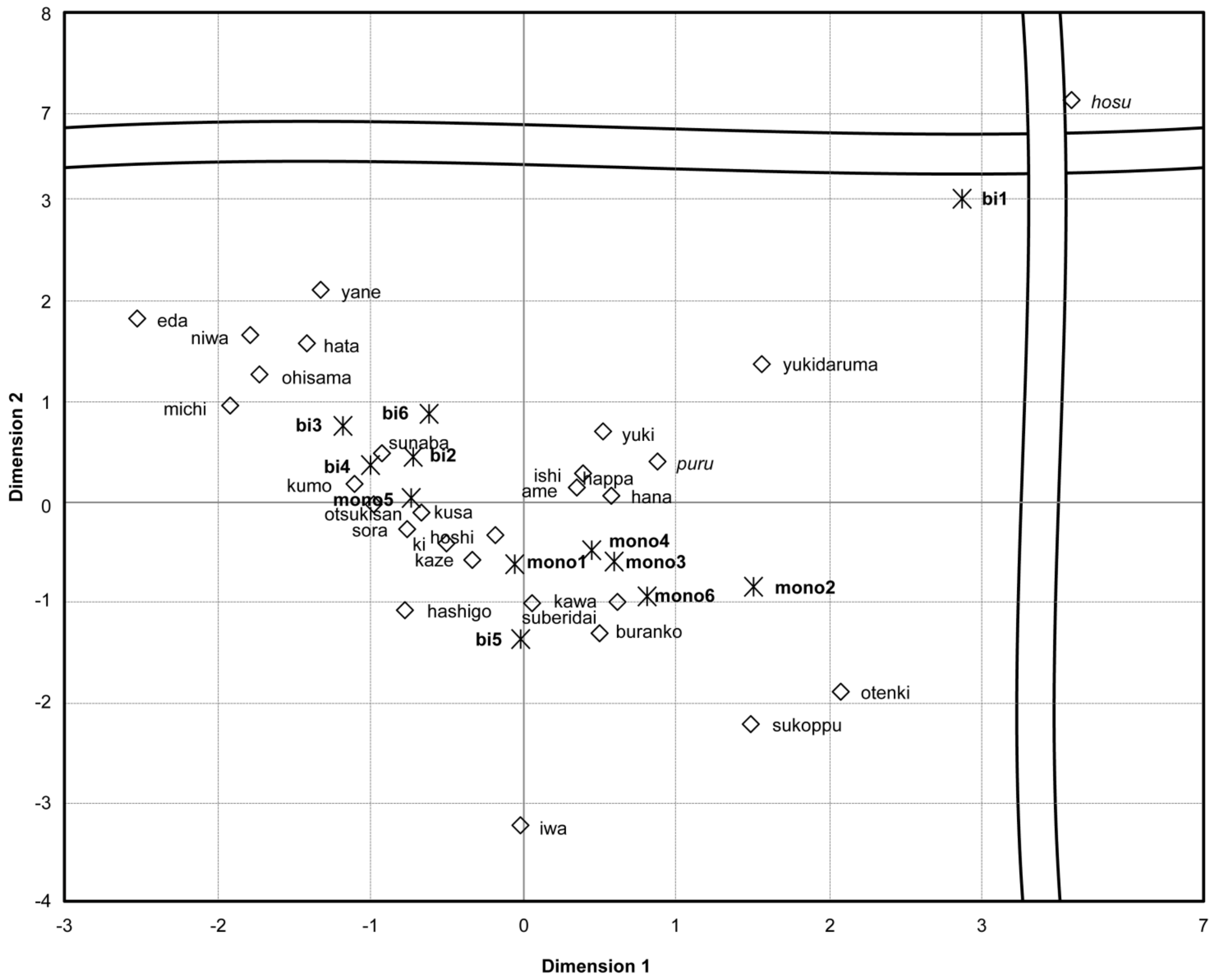

Figure S10. Characteristics of productive words in category K (places to go) for bilinguals and monolinguals
